# A randomized, double-blind sham-controlled trial on the efficacy of arthroscopic tennis elbow release for the management of chronic lateral epicondylitis

**DOI:** 10.1186/s12891-016-1093-9

**Published:** 2016-06-01

**Authors:** Bogdan A. Matache, Randa Berdusco, Franco Momoli, Peter L. C. Lapner, J. W. Pollock

**Affiliations:** Division of Orthopaedic Surgery, University of Ottawa, Ottawa, Ontario Canada; Orthopedic Shoulder, Knee and Sports Injuries, Pan Am Clinic, University of Manitoba, Manitoba, Canada; School of Epidemiology, Public Health, and Preventive Medicine, University of Ottawa, Ottawa, Ontario Canada; Ottawa Hospital Research Institute, Ottawa, Canada; Children’s Hospital of Eastern Ontario Research Institute, Ottawa, Canada

**Keywords:** Lateral epicondylitis, Tennis elbow, Arthroscopic tennis elbow release, Randomized controlled trial

## Abstract

**Background:**

Tennis elbow is a common elbow pathology typically affecting middle-aged individuals that can lead to significant disability. Most cases resolve within 2 years of symptom onset, but a subset of patients will develop persistent symptoms despite appropriate conservative management. There are several surgical approaches used to treat chronic tennis elbow, with arthroscopic surgery becoming an increasingly popular approach to address this pathology in North America. This procedure involves the arthroscopic release of the extensor carpi radialis brevis tendon (ECRB) origin at the elbow. The potential benefit of arthroscopic treatment of this condition is improved patient outcomes and shorter recovery time following surgery. The results of this technique have been reported only in the context of case series, which have shown positive results. However, in order to justify its widespread use and growing popularity, a high level of evidence study is required. The purpose of this prospective, randomized sham-controlled trial is to determine whether arthroscopic tennis elbow release is effective at treating chronic lateral epicondylitis.

**Methods:**

We will conduct a prospective single center, double-blind, randomized sham-controlled parallel arm trial evaluating the efficacy of arthroscopic tennis elbow release in adult patients with symptoms for at least 6 months. Patients will undergo intraoperative randomization after diagnostic arthroscopy of the elbow to receive either ECRB release (through the creation of a lateral portal) or a sham lateral portal and no ECRB release. The primary outcome will be the Mayo Elbow Performance Score (MEPS) at 1 year follow-up. Secondary outcomes will be the abbreviated Disability of the Shoulder and Hand (DASH) score, American Shoulder and Elbow Surgeons elbow (ASES-e) score and grip strength at 3, 6, 12 and 24 months as well as return-to-work time, ability to return to full duty and adverse outcomes.

**Discussion:**

Results of this study will provide empirical high quality evidence to guide clinical decision-making in patients with chronic tennis elbow.

**Trial registration:**

NCT02236689 (September 8, 2014)

## Background

Lateral epicondylitis (tennis elbow) is a common occurrence in the general population with an incidence of 4-7/1000/year [1–3]. More recent literature describes a 1–3 % rate over the course of a lifetime, most typically affecting individuals between the ages of 35 and 50 [4]. Despite its name, this condition affects a wide variety of individuals, including politicians, municipal utility employees, cooks, meat industry employees as well as nonlabor workers. One study looking at the financial burden of elbow epicondylitis in Washington State from 1987 to 1995 found that it accounted for 11.7 % of work-related injury claims, costing $6,593 per case in average direct workers’ compensation [5].

Although tennis elbow can present acutely, the onset is often insidious secondary to repetitive wrist extension and alternating forearm pro-supination. Symptoms include lateral elbow pain and forearm weakness that is exacerbated by repetitive extension and/or rotation of the wrist. Grip strength is also typically diminished. Its natural history is often reported as 6–24 months, [6–8] with more than 80 % of cases achieving complete resolution at 1 year [9–11]. However, some studies report a full recovery rate as low as 34 % by 12 months [12].

Non-operative treatment consists mainly of activity modification, nonsteroidal antiinflammatory medications, physical therapy, counterforce bracing and corticosteroid injection [13]. Many authors recommend at least 6 months of non-operative management before considering operative intervention [14, 15]. Despite these measures, some patients will develop chronic symptoms refractory to conservative care.

Surgical indications for the management of lateral epicondylitis include persistent pain and failed adequate conservative management. The goals of surgery are to directly address that area of pathology through a procedure that involves resection of the involved tissue, to stimulate neovascularization and to produce a healthy scar while doing the least possible damage to surrounding tissues [16]. Current surgical options can be classified into open, percutaneous and arthroscopic, with arthroscopic tennis elbow release (ATER) having gained popularity over the past fifteen years with improved understanding of three-dimensional elbow anatomy and advances in arthroscopic procedures and equipment. When performed by experienced specialists, arthroscopic surgery allows for the assessment and debridement of concomitant intra-articular pathology such as synovitis, radiocapitellar plicae, osteochondral defects and intra-articular loose bodies that are often missed and can be a frequent cause of residual pain following extensor carpi radialis brevis (ECRB) release [17–20]. For example, in a retrospective review of 36 patients treated arthroscopically for tennis elbow, 28 % had significant intra-articular synovitis requiring debridement [21]. The rehabilitation process and time back to work following arthroscopic treatment is faster in comparison to the other surgical approaches and the outcomes are generally reported as equal or better [17, 19, 22, 23]. A recent retrospective cohort study on 341 consecutive patients comparing arthroscopic to open release demonstrated significant differences in Disability of the Shoulder and Hand (DASH) scores and total number of excellent outcomes between groups, favoring arthroscopy [24].

Despite the increased popularity of ATER in recent years, there have been no randomized-controlled trials evaluating its efficacy. A recent systematic review concluded that there is fair-quality evidence for elbow arthroscopy in the treatment of lateral epicondylitis (grade B recommendation) on the basis that outcomes appear to be similar to open surgery [25]. This recommendation is based on two cohort studies (Level III) and eight case series (Level IV) all reporting good to excellent results for arthroscopic surgery. However, a subsequent Cochrane review concluded that due to the small number of studies, large heterogeneity in interventions across trials, small sample sizes and poor reporting of outcomes, there was insufficient evidence to support or refute the effectiveness of surgery for tennis elbow [26].

The arthroscopic approach to the treatment of lateral epicondylitis has been widely adopted in North America. However, no comparative studies have demonstrated its efficacy. In order to provide optimal care to patients and to justify the increased cost and utilization of resources required for this treatment, a high level of evidence study is essential. This study aims to elicit whether ATER is efficacious at treating chronic tennis elbow.

## Methods

### Study design

This study is designed as a prospective single center, double-blind, randomized sham-controlled parallel arm trial, stratified on the need for debridement. After diagnostic arthroscopy, patients will be randomly assigned to ATER or sham (i.e. placebo) surgery and stratified by whether they require debridement or not. The study is designed to abide by the current international research standards and will be reported according to the guidelines listed in the CONSORT statement [27]. The study is approved by the Ottawa Health Science Network Research Ethics Board of Ottawa, Canada and is in compliance with the Tri-Council Policy Statement: Ethical Conduct for Research Involving Humans; the International Conference on Harmonization – Good Clinical Practice: Consolidated Guideline and the provisions of the Personal Health Information Protection Act 2004.

The surgeries will be performed by a single fellowship-trained upper extremity orthopaedic surgeon with at least seven years of independent practice experience at a university-affiliated academic center.

### Purpose and hypothesis

The purpose of the study is to determine whether ATER is superior to non-operative management using a sham surgery control group for the management of chronic lateral epicondylitis of the elbow at 24 months postoperative as measured by the Mayo Elbow Performance Score (MEPS). Data will be collected at baseline and at 3, 6, 12 and 24 months following surgery. In addition, we will aim to determine if there is a differential effect of ATER versus sham surgery among the subgroup of patients requiring additional elbow debridement due to coexisting intra-articular pathology. Furthermore, we will compare the effect of ATER and sham surgery on the abbreviated DASH and American Shoulder and Elbow Surgeons Elbow (ASES-e) scores at the different timepoints, as well as assess for any differences in grip strength, return to work time and adverse outcomes between the two groups.

We hypothesize that at 24 months, the improvement in MEPS is greater after ATER than sham surgery. Furthermore, we hypothesize that the DASH, ASES-e will also favor ATER at all postoperative timepoints. In addition, we hypothesize that grip strength will be diminished in the ATER group compared to sham, but that return to work time will favor ATER Table 1.

**Table 1:** Data collection timeline

Variable	Baseline^a^Preoperative	T = 2 weeks Postoperative	T = 6 weeks Postoperative	T = 3 months Postoperative	T = 6 months Postoperative	T = 1 year^b^Postoperative	T = 2 years Postoperative
*Patient demographics*							
Age (years)	X						
Sex (M/F)	X						
Duration of symptoms (months)	X						
Arm affected (L/R)	X						
Dominant arm (L/R)	X						
Height (cm)	X						
Weight (kg)	X						
Body-Mass Index (BMI)	X						
Occupation	X						
Smoker (Y/N)	X						
Diabetic (Y/N)	X						
*Outcome measure*							
MEPS^c^	X			X	X	X	X
*Quick*DASH	X			X	X	X	X
ASES-e	X			X	X	X	X
Grip strength (grip dynamometer)	X			X	X	X	X
Return to work (Y/N)				X	X	X	X
Complications		X	X	X	X	X	X

^a^Second clinical visit

^b^Primary endpoint

^c^Primary outcome measure

### Participants

Patients eligible for inclusion in the study will be aged >18 years and have had symptoms for at least 6 months and have subsequently failed a 3-month course of conservative management consisting of activity modification, anti-inflammatories, physiotherapy, corticosteroid and platelet-rich plasma injections. Patients with an alternative diagnosis that better explains their symptoms or those who have had significant prior elbow trauma or surgery are not eligible to be a part of the trial. Workplace Safety and Insurance Board (WSIB) patients (workplace injuries) are also excluded from the study. Patients must be able to speak English. General Practitioners in the Ottawa area will be sent a letter asking them to refer patients who meet the eligibility criteria listed above. Eligible patients will receive informed consent on the two treatment arms. Enrolled participants will undergo a functional outcome scores assessment (MEPS, abbreviated DASH, ASES-e, grip strength) at the second preoperative clinical visit, which will serve as a baseline for comparison. They will then be scheduled for surgery through the standard outpatient surgery program.

### Randomization

The patients will be brought to the operating room where they will undergo a regional block anesthetic. A diagnostic arthroscopic evaluation will then be performed through a medial portal. Following this, the surgeon will make a decision as to whether the elbow requires any debridement. The decision to debride is a clinical one and is influenced by the presence of an intra-articular pathology, such as extensive synovitis (inflammation), loose bodies and radiocapitellar plicae. Patients who are deemed to require intra-articular debridement will receive it at this point. This requires the creation of a lateral, working portal. Patients who do not require debridement will be randomized to ATER or sham. Similarly, patients who do require debridement will be randomized to ATER or sham.

**Fig. 1:**
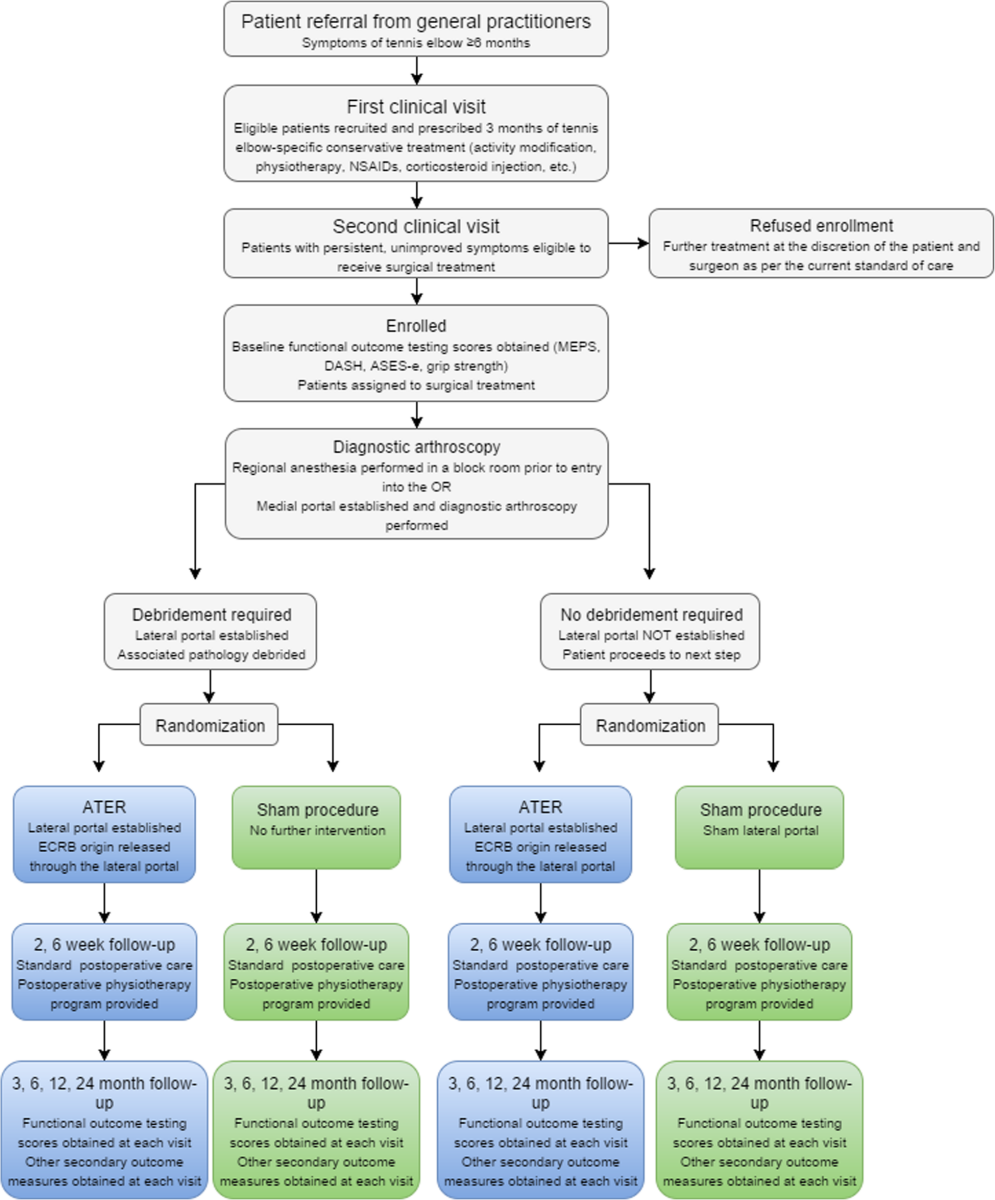
Study flowchart. Legend: two parallel randomization arms; ATER (*blue*) and sham procedure (*green*)

A randomization sequence will be computer generated by an independent statistician. Consecutive patients will be randomized to receive ATER or Sham surgery in a 1:1 allocation ratio, with stratification by the need for debridement and randomly permuted block sizes from 2, 4, or 6. Central randomization will be accomplished by the use of online registration and verification of eligibility through the Data Management Services group at the Ottawa Hospital Research Institute Fig. 1.

### ECRB release

The patient is positioned in the lateral position with the body supported using a beanbag and the arm over a right-angle arm support. They are padded and protected appropriately. The patient’s bed is tilted towards the surgeon approximately 15° to avoid any traction on the brachial plexus. The patient is then prepped and draped in the standard sterile fashion. Anatomic landmarks are identified and marked using a marking pen. All portal sites are determined and marked. The elbow is injected with 25 mL of normal saline to provide capsular distention and safer portal placement.

The anteromedial portal is established first. This is done with a blunt trocar. The arthroscope is then inserted into the elbow. The anterolateral portal is then established under direct visualization. This is also completed with a blunt switch stick. A 6 mm dilator is then placed over the switch stick.

Next, a shaver is used to remove the lateral capsule in order to visualize the ECRB. A wedge of the capsule in that region is removed. We are careful to avoid an anterolateral capsulectomy due to the proximity of the posterior interosseus nerve in this location. We are also careful during the procedure to avoid disrupting the ulnar lateral collateral ligament by staying anterior to the equator of the capitellum and radial head. Once the ECRB is visualized, a biting forceps is used to release the tendon. The extensor carpi radialis longus (ECRL) is left intact. Finally, a 1.5 × 1.5 × 1.5 triangular section of the ECRB tendon (and superior edge of the Extensor Digitorum Communis) origin is excised. The ECRL and LUCL are left intact. A complete release of ECRB is performed in order to obtain good visualization of the overlying ECRL.

After the release is completed, the elbow is examined to demonstrate full, smooth, concentric range of motion and to confirm that there is no evidence of catching or clicking. The elbow is examined with varus, valgus, rotational and axial stress to confirm that the LCL and MCL are intact.

### ATER: Intervention

Patients randomized to ATER will receive arthroscopic release of the origin of the ECRB tendon through a standard, two-portal arthroscopic technique.

### Sham surgery: Control

Patients randomized to sham surgery will not have the ECRB muscle origin released. Instead, they may receive a small incision only through the skin in the same position where a second lateral portal would otherwise be introduced, depending on whether or not they required elbow debridement in the prior step. If debridement was previously performed, the lateral portal will already have been created. If no debridement was required, they will receive a sham lateral portal, thus minimizing the risks associated with introducing a second true portal while also preserving the patient’s blinded status.

### Postoperative protocol

Post-operative follow-up is at 2 weeks, 6 weeks, 3, 6, 12 and 24 months. Functional outcome scores will be obtained from each patient at the 3, 6, 12 and 24 month time points. Post-operative management will be identical among the two treatment arms and consists of immediate elbow range of motion exercises and no weight-bearing restrictions. However, elbow, forearm and shoulder strengthening exercises will begin at 10 weeks postoperative. Patients (independent of concealed group allocation) will receive functional counseling by a licenced physiotherapist at the first postoperative visit and will be provided with a standardized exercise protocol to guide outpatient physiotherapy.

### Primary outcome

#### MEPS

All functional assessment forms will be administered electronically to patients by means of a touch-screen device at each follow-up appointment. Our primary outcome measure is elbow pain and function as measured by the MEPS at 24 months post-operative. The MEPS is a commonly used performance index for evaluation of clinical outcomes for a variety of elbow disorders that can be completely patient-administered [28]. It consists of four parts: pain, stability, ulnohumeral motion and the ability to perform five functional tasks. The highest possible score is 100, with scores 90 and above indicating excellent function [29].

### Secondary outcomes

#### DASH

The abbreviated (quick) DASH score is a self-reported questionnaire consisting of 11 items designed to measure symptoms and function in patients with upper extremity-specific pathologies. It is a shortened version of the DASH score, which normally consists of 30 items, and has been shown to retain its psychometric properties in a more user-friendly structure. At least 10 of the 11 items must be completed in order for the score to be valid. Each item is scored 1-5, with 1 indicating no difficulty and 5 indicating inability to perform the function questioned. The values are then summed up, averaged and converted to a score out of 100 by subtracting 1 and multiplying by 25. The higher the score, the greater the disability [30].

#### ASES-e

The ASES-e score is both patient and physician-administered. The patient-reported self-evaluation consists of pain, function and satisfaction components. The pain component utilizes the visual analogue scale (0 to 10, with 10 indicating the worst pain ever), while the function component pertains to the patient’s ability to perform specific upper extremity-related tasks (0 to 4, with 4 indicating no difficulty). Satisfaction is graded 0 to 10. The physician assessment consists of motion, strength (both in flexion, extension, pro-supination), stability (varus, valgus, posterolateral rotatory) and physical findings (tenderness, scars, etc). Stability is graded 0 to 3, with 3 grading gross instability, while strength is graded 0 to 5, with 5 indicating normal strength. Physical findings are reported as being present or not.

The MEPS, DASH and ASES-e scores are all validated, upper extremity-specific questionnaires [29]. We will also be testing grip strength using a standard grip dynamometer and compare return to work time and adverse outcomes between the two groups.

### Blinding

Patients will be blinded to treatment group through the use of a sham incision. Due to the nature of a surgical trial, the surgeon cannot be blinded to the intervention. However, a trained independent assessor, blinded to treatment status will conduct the follow-up examinations in a standardized fashion. This will minimize the potential for biases introduced by the examiner when performing the physical assessment and recording data. The assessor will not have access to the patient chart prior to the examination. To help reduce the potential for observer bias, the physical examination and the administration of study questionnaires are standardized.

### Control of contamination and co-intervention

There will be a possibility of cross-over after one year postoperative. This means that to remain a study participant, the patient may not learn which of the two groups they were randomized to until one year postoperative. However, should the patient be unsatisfied with their outcome prior to that mark, they may elect to withdraw themselves from the study and learn which treatment they received and pursue further treatment if appropriate.

### Ensuring complete follow-up

The following measures will help to ensure completion of follow-up: a) study patients will supply their own address, telephone and email information as well as the name, relationship, address, telephone and email information of someone who does not reside with the patient, but is likely to have contact with them should they move or change telephone information, b) patients will receive a copy of the consent form which will outline the purpose of the study, the importance of their individual participation and attendance for follow-up assessments and c) the research coordinator will maintain regular contact with the patients.

### Statistical considerations

#### Primary analysis

The primary analysis involves a comparison of the MEPS outcome measure between the two surgical treatment groups on an intention-to-treat basis at 24 months post-surgery as compared to baseline values. A two-sample independent ANCOVA will be used to assess whether there is a statistically significant difference between groups for the MEPS scores, accounting for the debridement stratification variable and baseline MEPS. Linear regression will be used to estimate treatment size effect, accounting for the stratification variable and baseline MEPS, as well as any possible imbalanced covariates. Primary analyses will be repeated for the debridement and no-debridement patient subgroups. Missing follow-up data will be addressed with multiple imputation techniques.

#### Secondary analysis

A generalized estimated equations (GEE) analysis will be conducted to determine whether there is an effect of treatment arm over time (repeated measures) on the MEPS outcome, considering measures taken preoperatively, and at 3, 6, 12 and 24 month follow-ups. Other secondary analyses involves a comparison of the secondary outcome measures between the two surgical treatment groups. Differences in the mean abbreviated DASH and ASES-e scores, as well as grip strength, will be tested with ANOVAs and effect sizes estimated with linear regression. Median time back to work in each group will be derived with Kaplan-Meier survival curves, and a statistical comparison between groups will be tested using a log rank test.

#### Sample size calculation

The primary outcome is the modified Mayo Elbow Performance score. A minimum clinically importance difference of 15 points has been derived for patients with rheumatoid arthritis [31]. In a study of patients with elbow dysfunction, standard deviations ranged from 17 to 19 points [28]. In order to attain a study power of 80 % to detect a minimally clinically important difference of 15 points on the MEPS primary outcome measure at one year follow-up using ANOVA to compare ATER versus sham, and assuming a standard deviation of 19 points as well as an alpha level of 0.05, a total sample size of 52 patients will be required (group sizes of 26) [31, 32]. However, adjusting for the remote possibility of 5 % of patients requiring un-blinding in the case of sham surgery (drop out), and a possible 15 % loss to follow-up yields a total sample size of 68 (34 in each group).

Assuming a recruitment and consent rate of 70 %, we would need to approach 97 patients to achieve our required sample size. Hospital records indicate that our clinic sees approximately 10 eligible patients per month, meaning we can feasibly achieve our required sample size within 10 months of enrollment.

Patients who are excluded from the trial intra-operatively and patients whose symptoms resolve prior to intra-operative randomization will only be followed clinically and not as part of this study.

## Discussion

We would like to stress the importance of considering risk when attempting to answer a clinical question. In any surgical study, participants are subjected to the possibility of suffering an adverse event and our proposed trial is no different. Thus, it is imperative from an ethical point of view to answer the following questions: 1) is the question worth asking, 2) is there an alternative, less risky approach that adequately answers the question, and 3) are the risks justified by the expected benefits of answering the question? The first point was previously addressed in our discussion about the rate of affliction of tennis elbow and the lack of evidence to support the efficacy of a commonly performed orthopaedic procedure aimed at treating it. We will thus focus our attention to points 2 and 3.

We strongly considered the alternative of having a control arm consisting of conservative management instead of diagnostic arthroscopy, as this would have been a less invasive option. However, a trial of this design would fail to further advance our understanding of the efficacy of ATER for the following reasons. Firstly, outcome raters could foreseeably become un-blinded if the patient communicates that they had surgery, as was previously shown to have occurred in a study of surgery versus splinting for carpal tunnel syndrome [33]. Second, there is a significant potential for recall bias given the outcome measures used. While the MEPS, abbreviated DASH and ASES-e questionnaires are validated scales for data acquisition, thereby lowering the risk of recall bias, they are nevertheless based on patient-reported data and are therefore at risk of bias [33]. Furthermore, patients included in the study would have already attempted and failed appropriate conservative management. Therefore subjecting them to the same failed therapy for a period of time exceeding the expected natural history of the disease would be of no added benefit to them.

Although patients randomized to sham surgery are not expected to directly benefit from their procedure, the diagnostic arthroscopy they would have received prior to intra-operative randomization is therapeutic in the sense that it allows for assessment and treatment of potential concomitant pathology, which may be present. A trial design of ATER versus non-operative management would prevent us from performing this assessment.

When looking at the complications of arthroscopic elbow surgery, it is important to consider the different pathologies separately as each one carries a different risk quotient. Elbow arthroscopy performed for the treatment of osteoarthritis, inflammatory arthritis and loose body removal has about a 0.8 % rate of joint infection [34]. Furthermore, a recent retrospective review of 1004 elbow arthroscopy procedures identified a transient neurologic deficit in 4 patients (2 ulnar, 1 radial nerve palsies), all of whom had complete resolution of symptoms by 6 weeks postoperative, and one permanent ulnar nerve palsy secondary to direct injury to the nerve (discovered on subsequent exploratory surgery) [35]. Conversely, arthroscopic surgery specifically for tennis elbow has an extremely low risk of complications. In a case-control series of 225 elbows treated arthroscopically for tennis elbow, there were no major complications such as deep infection, permanent nerve injuries or elbow stiffness [24]. Thus, the primary burden of undergoing this procedure is pain at the surgical site. However, pain from arthroscopic surgery is generally low-grade and well-tolerated by patients. We believe that the risks of our proposed study are outweighed by the expected benefit of being able to definitely answer whether arthroscopic tennis elbow release efficaciously treats the common pathology of chronic lateral epicondylitis.

## Abbreviations

ANCOVA, analysis of covariance; ANOVA, analysis of variance; ASES-e, American Shoulder and Elbow Surgeons – elbow; ATER, arthroscopic tennis elbow release; CONSORT, consolidated standards of reporting trials; DASH, disability of the hand and shoulder; ECRB, extensor carpi radialis brevis; GEE, generalized estimated equations; MEPS, Mayo elbow performance score; WSIB, Workplace Safety and Insurance Board.
